# Immunolocalization of Sphingolipid Catabolism Enzymes along the Nephron: Novel Early Urinary Biomarkers of Renal Damage

**DOI:** 10.3390/ijms242316633

**Published:** 2023-11-23

**Authors:** Martha Franco, Agustina Cano-Martínez, María del Pilar Ramos-Godínez, Rebeca López-Marure, Luis Donis-Maturano, José Santamaría Sosa, Rocio Bautista-Pérez

**Affiliations:** 1 Department of Cardio-Renal Pathophysiology, Instituto Nacional de Cardiología “Ignacio Chávez”, Mexico City 14080, Mexico; marthafranco@lycos.com (M.F.);; 2 Department of Physiology, Instituto Nacional de Cardiología “Ignacio Chávez”, Mexico City 14080, Mexico; agustina.cano@cardiologia.org.mx (A.C.-M.); rebeca.lopez@cardiologia.org.mx (R.L.-M.); 3 Department of Electron Microscopy, Instituto Nacional de Cancerología, Mexico City 14080, Mexico; pilyrg@gmail.com; 4 Faculty of Higher Studies Iztacala, Universidad Nacional Autónoma de México, Mexico City 54090, Mexico; ludoma6@hotmail.com; 5 Department of Molecular Biology, Instituto Nacional de Cardiología “Ignacio Chávez”, Mexico City 14080, Mexico

**Keywords:** sphingomyelinase, ceramidase, sphingosine kinase, urine biomarker, diabetes, angiotensin II-induced hypertension

## Abstract

The objective of this study was to investigate whether the activity of enzymes involved in sphingolipid catabolism could be biomarkers to predict early renal damage in streptozotocin (STZ)-induced diabetic rats and Angiotensin II (Ang II)-induced hypertension rats. Diabetic and hypertensive rats had no changes in plasma creatinine concentration. However, transmission electron microscopy (TEM) analysis showed slight ultrastructural changes in the glomeruli and tubular epithelial cells from diabetic and hypertensive rats. Our results show that the acid sphingomyelinase (aSMase) and neutral sphingomyelinase (nSMase) activity increased in the urine of diabetic rats and decreased in hypertensive rats. Only neutral ceramidase (nCDase) activity increased in the urine of diabetic rats. Furthermore, the immunofluorescence demonstrated positive staining for the nSMase, nCDase, and sphingosine kinase (SphK1) in glomerular mesangial cells, proximal tubule, ascending thin limb of the loop of Henle, thick ascending limb of Henle’s loop, and principal cells of the collecting duct in the kidney. In conclusion, our results suggest that aSMase and nCDase activity in urine could be a novel predictor of early slight ultrastructural changes in the nephron, aSMase and nCDase as glomerular injury biomarkers, and nSMase as a tubular injury biomarker in diabetic and hypertensive rats.

## 1. Introduction

In metabolic, cardiovascular, and renal diseases, the cellular content of the sphingolipids and their metabolites are regulated by enzymes involved in the anabolism (glucosylceramide synthase, sphingomyelin synthase, ceramide synthase) and catabolism (SMase, CDase, SphK, and sphingosine-1-phosphate lyase). In the kidney and other organs, the expression or activity of these enzymes changes in pathophysiological conditions [1,2,3,4,5,6,7,8,9]. These findings are important since the failure of one organ can induce pathological changes in another organ, or ambos may present simultaneous dysfunction (e.g., Kidney–heart, kidney–liver) [10,11].

In the kidney, there is nSMase expression in principal and intercalated cells of the collecting duct, and its activity is stimulated by fluid shear stress [12]. The nCDase is localizing at the apical membrane of tubules [13]. SphK1 is expressed in the cortex, followed by the medulla and papilla [14,15]. In addition, SphK1 and SphK2 are expressed throughout development, predominantly in the metanephric mesenchyme [16]. 

During development and aging, the nSMase and CDase activity increases in the kidney [17]. In the renal cortical of serum nephritis rats and rats intoxicated with carbon tetrachloride, SMase activity increased. In contrast, after renal injury by ischemia, ischemic/reperfusion, or myohemoglobinuric, SMase activity decreased [18,19]. After the induction of diabetes, nCDase and SphK1 activity increased, and only nCDase activity decreased after 28 days [20]. In the kidneys of diabetic rats, nSMase activity increased, and nCDase activity decreased, whereas in the kidney of hypertensive rats, the activity of both enzymes increased [9].

These enzymes, sphingolipids, and their metabolites can be detected in blood or urine, so they have been proposed as biomarkers of kidney damage. In this regard, in the plasma of patients or preclinical animal models, the enzymatic activity, the concentration of sphingolipids, and their metabolites have already been determined [6,9,21,22,23]. Interestingly, in the urine of diabetic patients, glycosphingolipids, ceramides, and hexose ceramide levels increase [24,25,26]. In patients with severe COVID-19, increased urinary levels of sphingomyelin, ceramides, sphingosine, and glycerophospholipids might result in renal damage [27]. Also, aSMase and nSMase were purified from human urine [28,29].

These findings demonstrate that sphingolipids, their metabolites, and enzymes involved in sphingolipid metabolism can be detected in the urine and are attractive candidates as biomarkers of renal damage. 

The objective of this study was to investigate whether the activity of enzymes involved in sphingolipid catabolism could be biomarkers to predict early renal damage in STZ-induced diabetic rats and Ang II-induced hypertension rats.

## 2. Results

### 2.1. General Characteristics of Diabetic and Hypertensive Rats

The blood glucose concentration was higher in diabetic rats compared with control rats (589 ± 34 vs. 113 ± 2.5 mg/dL). The systolic blood pressure (SBP) was elevated in hypertensive rats compared with normotensive rats on day 14 (213.4 ± 4 vs. 125.9 ± 2.5 mm Hg). The plasma creatinine concentration showed no changes in diabetic rats compared with control rats (0.56 ± 0.06 mg/dL vs. 0.5059 ± 0.07 mg/dL); the hypertensive rats also showed no changes (0.471 ± 0.1496 mg/dL vs. 0.4664 ± 0.15 mg/dL).

### 2.2. Transmission Electron Microscopy (TEM)

**Figure 1:**
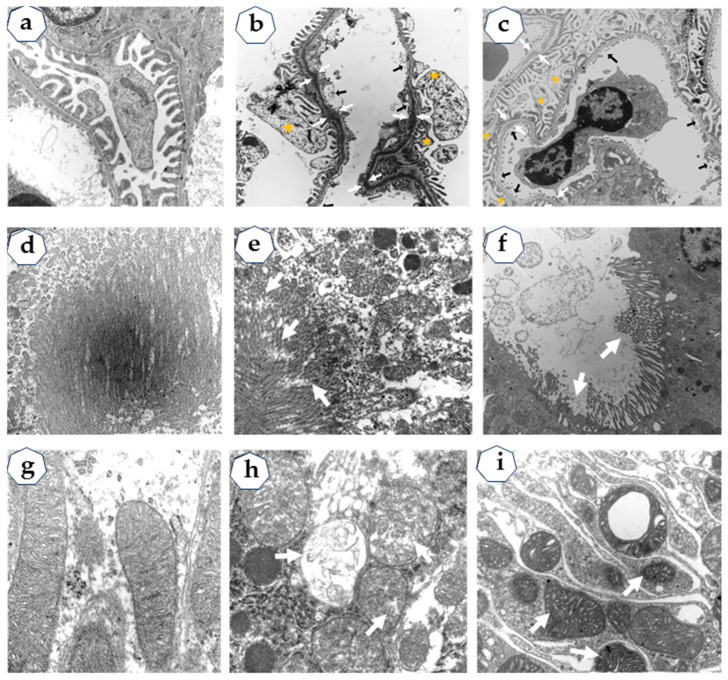
Representative electron micrographs of kidney from control (**a**,**d**,**g**), diabetic (**b**,**e**,**h**) and hypertension rats (**c**,**f**,**i**). Glomeruli: (**a**) detached endothelial layer in the glomerular capillary (thick arrow), glomerular basement membrane thickening (GBM) (arrow pairs), and fusion of podocytes foot (stars) (**b**,**c**). The proximal tubule shows that decreased and irregular microvilli (**e**,**f**), the mitochondria are round with loss of matrix density (**h**,**i**), and swelled cristae (**h**,**i**). Supplemental material available online with this article.

TEM analysis showed slight ultrastructural changes in the kidney from diabetic and hypertension rats: disrupted glomerular ultrastructure (detached endothelial layer in the glomerular capillary, glomerular basement membrane thickening, effacement of podocytes, and fusion of podocytes foot processes; the tubular epithelial cells show decreased and irregular microvilli, and the mitochondria are round with loss of matrix density (Figure 1). 

### 2.3. Sphingomielinase and Ceramidase Activity in Urine of Diabetic and Hypertensive Rats

**Figure 2:**
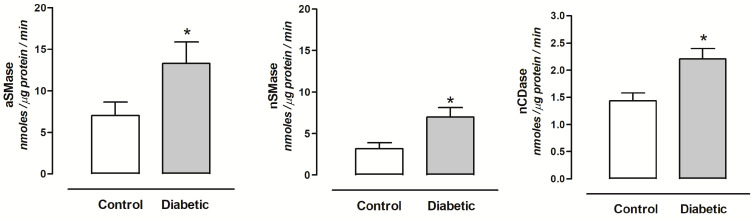
Enzymatic activity of aSMase, nSMase, and nCDase in the urine of control, and diabetic rats. Each bar represents the mean ± SE of *n* = 10. * *p* < 0.05 diabetic vs. control rats.

In the urine of diabetic rats, the aSMase and nSMase activity was increased, while the aCDase activity did not show changes, and nCDase activity also increased compared with the control group (Figure 2).

**Figure 3:**
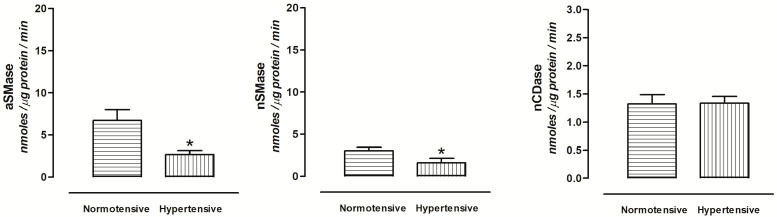
Enzymatic activity of aSMase, nSMase, and nCDase in the urine of normotensive, and hypertensive rats. Each bar represents the mean ± SE of *n* = 10. * *p* < 0.05 hypertensive vs. normotensive rats.

In the urine of hypertensive rats, the aSMase and nSMase activity decreased, while the aCDase and nCDase activity did not show changes compared with the normotensive group (Figure 3). Moreover, the nSMase activity was lower compared to aSMase activity in both groups. 

### 2.4. Expression and Localization of Enzymes Involved in Sphingolipid Catabolism along the Nephron

**Figure 4:**
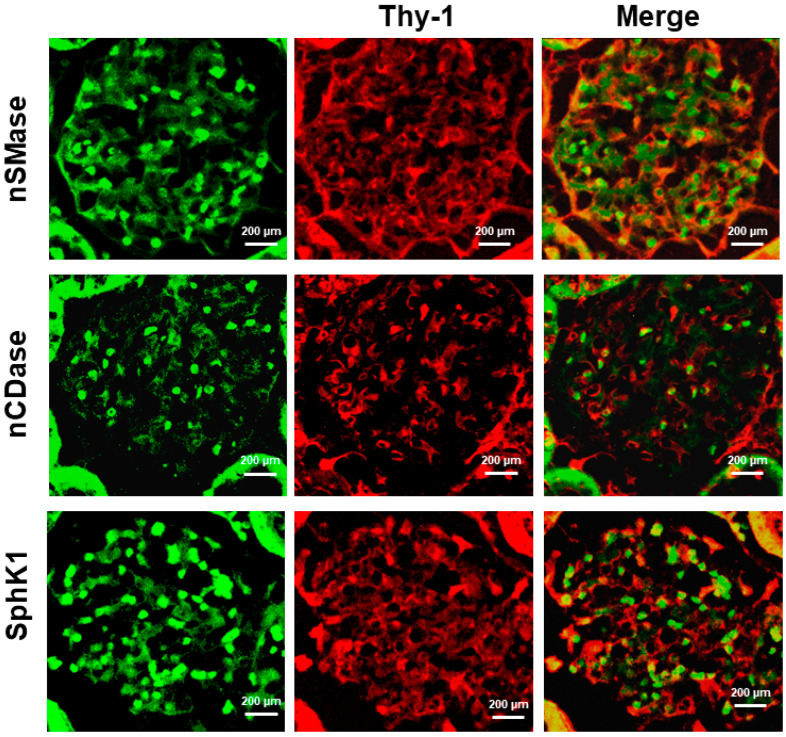
Coimmunostaining of nSMase, nCDase or SphK1 with Thy-1 as marker of mesangial cells in kidney.

The functional unit of the kidney is the nephron; each nephron consists of glomerulus, proximal tubule (convoluted and straight segments), loop of Henle, distal tubule, the connecting tubule, and collecting duct. The glomerulus is formed of three types of cells: endothelial cells, podocytes, and mesangial cells. In the kidney of control, diabetic, and hypertensive rats, we observed expression of all markers. However, we only show the photographs of the control group. As shown in Figure 4, Thy-1 is a mesangial cell marker, and these cells were also positive for nSMase, nCDase, and SphK1.

**Figure 5:**
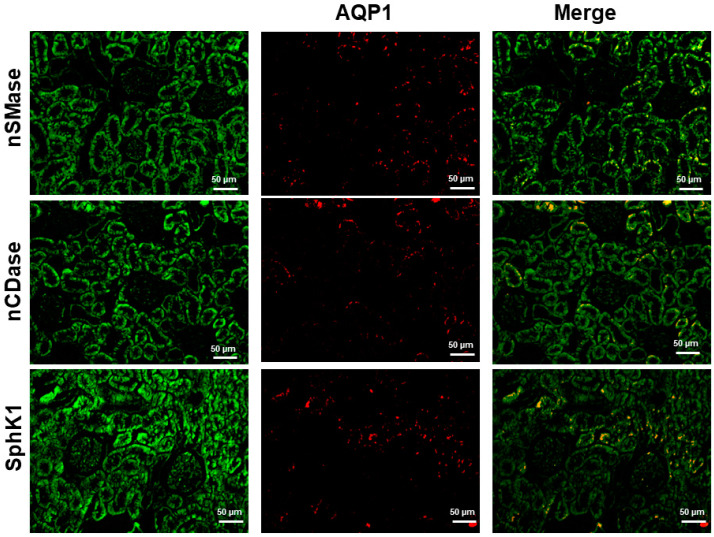
Coimmunostaining of nSMase, nCDase or SphK1 with AQP1 as a marker of proximal tubule in kidney.

The proximal tubule is responsible for 70% of water reabsorption and descending thin limb of Henle’s loop by 20%; the aquaporin-1 (AQP1) facilitates water transport across the membranes’ apical and basolateral membranes in these two nephron segments. In this study, AQP1 was used as a marker of the proximal tubule. As shown in Figure 5, nSMase, nCDase, and SphK1 were also immunolocalized to proximal tubule cells. 

**Figure 6:**
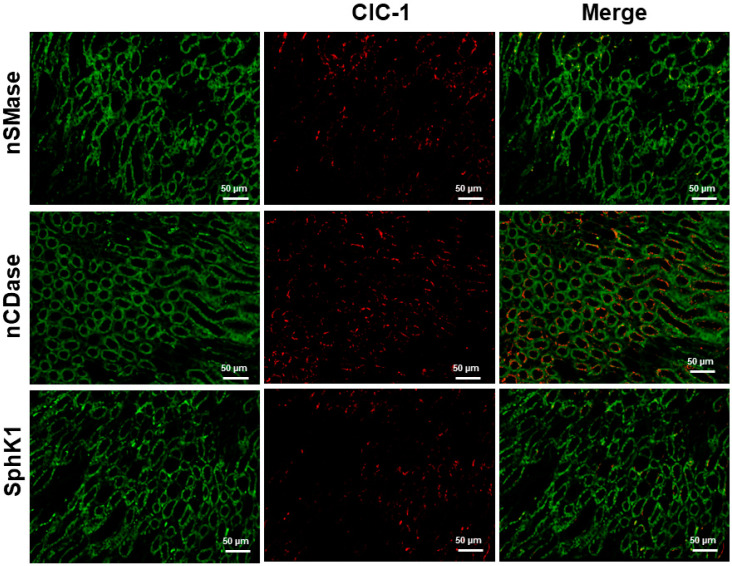
Coimmunostaining of nSMase, nCDase or SphK1 with CIC-1 as a marker of ascending thin limb of the loop of Henle in kidney.

The ascending thin limb expressed the Cl^−^ channel ClCK1 along the entire lengths and was used as a marker of this nephron segment. As shown in Figure 6, nSMase, nCDase, and SphK1 were also immunolocalized to the ascending thin limb of the loop of Henle. 

**Figure 7:**
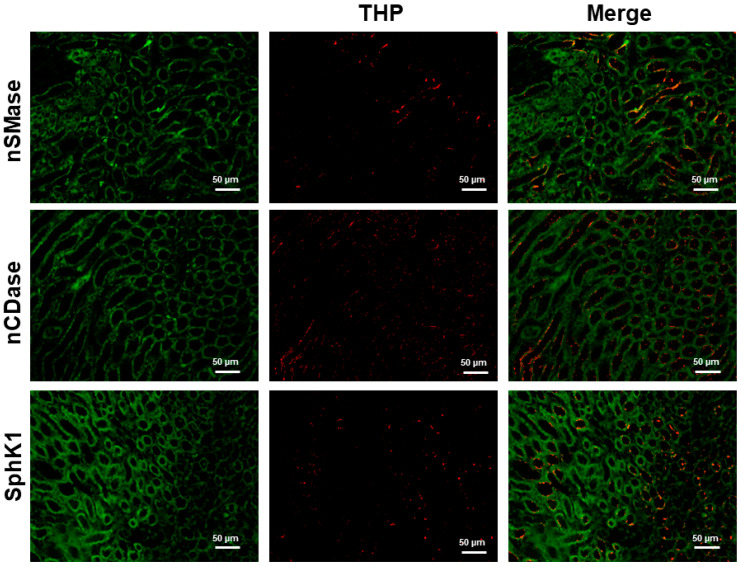
Coimmunostaining of nSMase, nCDase or SphK1 with THP as a marker of ascending thick limb of Henle’s loop (TAL) in kidney.

The Tamm–Horsfall (THP) or uromodulin protein is expressed in the ascending thick limb of the loop of Henle, extending through the whole outer medulla and cortex. As shown in Figure 7, THP colocalized with nSMase, nCDase, and SphK1 in the ascending thick limb of the loop of Henle. 

The distal convoluted tubule, the connective tubule, and the collecting duct form the distal part of the nephron and oversee the final tuning of renal excretion. 

**Figure 8:**
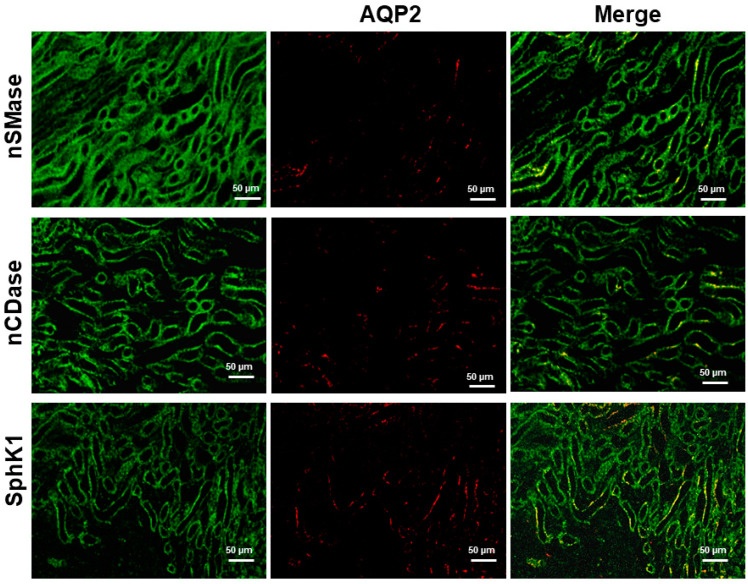
Coimmunostaining of nSMase, nCDase or SphK1 with AQP2 as a marker of principal cells of the collecting duct (CD) in kidney.

In the collecting duct, two functionally and morphologically distinct cells are present: principal cells and intercalated cells. The primary mechanism by which water is reabsorbed in the principal cell is through aquaporin 2 (AQP2). As shown in Figure 8, AQP2 colocalized with nSMase, nCDase, and SphK1 in the principal cells. 

## 3. Discussion

In this study, we evaluated whether the activity of enzymes involved in sphingolipid catabolism could be biomarkers to predict early renal damage in diabetic and hypertensive rats. Diabetic and hypertensive rats had no changes in plasma creatinine concentration. However, TEM analysis showed slight ultrastructural changes in the glomeruli and tubular epithelial cells.

Our results agree with studies that using serum creatinine concentration-based criteria does not correlate well with the severity of renal injury. The serum creatinine can increase in the absence of glomerular or tubular injury and can be unchanged under conditions of glomerular or tubular injury [30,31]. Thus, it is necessary to find specific and sensitive biomarkers that reflect a pathophysiological process, and these can easily be detected in the plasma or urine.

Urine is an ideal biomarker source for clinical metabolomics or proteomics studies [32]. The concentration of some sphingolipid metabolites increases in urine from diabetic patients and patients with severe COVID-19 [24,25,26,27]. Interestingly, aSMase and nSMase were purified from human urine [28,29]. Thus, the determination of urinary enzymes may be valuable for the diagnosis of renal damage [33]. 

We recently reported that the aSMase and nCDase activity increased in the plasma of diabetic and hypertensive rats [9]. This increase is possibly due to fact that interleukin 1β (IL-1β) and interferon-gamma (IFN-gamma) stimulate the secretion of aSMase in endothelial cells [34,35,36]. Thus, our results suggest that aSMase and nCDase are plasma proteins that cross the slightly damaged glomerular barrier and increase their elimination in the urine of diabetic rats, which explains their increase in activity.

On the other hand, some studies reported the localization of murine and human nSMase in the nucleus, endoplasmic reticulum, Golgi apparatus, lysosomes, mitochondria, and plasma membrane [37,38,39]. In this study, we show nSMase expression along the nephron, proximal tubule cell with decreased and irregular microvilli, and round mitochondria with a loss of matrix density, which may contribute to the release of nSMase in the urine of diabetic rats. 

In addition, an imbalance in the levels of sphingolipids or their metabolites results in changes in the structure and function of mitochondria, leading to the overproduction of reactive oxygen and nitrogen species [40]. The reactive oxygen/nitrogen species can activate aSMase and nSMase [41,42]. Also, the depletion of reduced glutathione and the concentration of magnesium (Mg^2+^) contribute to the regulation of nSMase activity [43,44]. Fluid shear stress also induces nSMase activity in principal cells and intercalated cells of the kidney [12]. These mechanisms explain why it increases the nSMase activity in the urine of diabetic rats. In contrast, aSMase activity decreased, while nCDase activity showed no changes in the urine of hypertensive rats. As mentioned above, the aSMase and nCDase are plasma proteins but do not cross the glomerular barrier because Ang II induces vasoconstriction and decreases the kidney glomerular filtration rate, as we have reported in previous studies [45,46]. 

On the other hand, the nSMase present in the urine of hypertensive rats may originate from kidney tissue; however, its activity decreases depending on the concentration of Mg [44]. Also, in renal mesangial cell cultures, the inflammatory cytokines, growth factors, and vasoconstrictor peptides regulate the SMase and CDase activity [47,48,49]. The bradykinin inhibits SMase activity in the cortical-collecting duct cells line [50].

In addition, it has been reported that dysregulation of sphingolipid metabolism in podocytes can compromise the proper functioning of the glomerular basement membrane, leading to proteinuria, glomerular fibrosis, and glomerulosclerosis [51]. Sphingosine-1-phosphate induces tubule interstitial renal inflammation and fibrosis in diabetic nephropathy [52]. Interestingly, we reported an S1P content increase in the kidney of diabetic and hypertensive rats [9].

Thus, sphingolipid catabolic enzymes could be biomarkers to predict early kidney damage in pathology such as diabetes and hypertension. The aSMase and nCDase can be considered as glomerular injury biomarkers and nSMase as a tubular injury biomarker.

## 4. Materials and Methods

### 4.1. Animal Models

Animal procedures were performed in accordance with the Mexican Federal Regulation for Animal Experimentation and Care (NOM-062-ZOO-2001), and protocols were approved by the Investigation Committee of the Instituto Nacional de Cardiología “Ignacio Chávez” (INC-CICUAL/012/2019, INC-CICUAL/005/2020). All rats had free access to water and standard chow diet.

#### 4.1.1. Streptozotocin-Induced Diabetic Rats

Male Wistar rats (300–350 g) were divided into two groups (*n* = 10 each): (1) control (injected with citrate buffer) and (2) streptozotocin (STZ)-induced diabetic rats (STZ, 65 mg/kg body weight). Blood glucose levels were measured at 24 h after STZ administration. The animals with blood glucose 300 mg/dL (18 mmol/L) were used as diabetic rats and maintained for 30 days [9]. 

#### 4.1.2. Angiotensin II-Induced Hypertension Rats

Male Wistar rats weighing 350–360 g were separated into two groups: normotensive (Sham, *n* = 10) and hypertensive (Ang II, *n* = 10) rats. Ang II (Sigma, St. Louis, MO, USA) was infused via subcutaneous osmotic minipumps (Alzet 2002; Alza, Palo Alto, CA, USA), implanted under isoflurane anesthesia. Minipumps delivered Ang II at a rate of 435 ng/kg/min. Experiments were performed 13–14 days after the beginning of Ang II infusion, as previously described [9,46].

#### 4.1.3. Sample Collection and Tissue Preparation

At 30 days after STZ administration or 2 weeks after the infusion of Ang II, urine samples were collected over a 24 h period and stored at −20 °C to assay for proteinuria by Bradford assay (Bio-Rad, Hercules, CA, USA), as well as to determine the activity of nSMase, acid SMase (aSMase), nCDase, and acid CDase (aCDase). 

The animals were anesthetized with sodium pentobarbital (30 mg/kg, i.p.); blood samples were taken and collected in heparinized tubes, and after centrifugation (10,000× *g* for 5 min), plasma was obtained. Plasma samples were stored at −20 °C and used for creatinine determination (Quanti Chrom™ creatinine assay kit, BioAssay Systems, Hayward, CA, USA) [53]. 

After decapsulation, the kidneys were perfused with ice-cold phosphate-buffered solution (PBS) pH 7.4, to remove red blood cells and clots. After perfusion with PBS, left kidney was sectioned in small pieces and embedded in Epon 812 for transmission electron microscopy (TEM). The right kidney was fixed in 4% paraformaldehyde-PBS, embedded in paraffin, and mounted in coverslips for immunostaining.

#### 4.1.4. Transmission Electron Microscopy (TEM) 

The kidneys were excised and fixed in PBS with 2.5% glutaraldehyde plus and 2.5% paraformaldehyde. The tissues were post-fixed with 1% osmium tetroxide (OsO4). Then, the fixed renal tissue fragments were dehydrated in an ascending series of ethanol and embedded in Epon 812 epoxy resin (sigma). Subsequently, the tissue was treated with uranyl acetate and incubated in a lead citrate solution. Samples were observed in a JEOL 10-10 transmission electron microscope (JEOL, Tokyo, Japan) [54].

### 4.2. Sphingomielinase Activity

The aSMase and nSMase activity was measured as previously described [9]. Fluorescence was measured at ƛ_ex_ = 545 nm and λ_em_ = 590 nm using a microplate reader (Synergy^®^ HTX multimode). 

### 4.3. Ceramidase Activity

aCDase and nCDase activity was measured as previously described [9]. Fluorescence was measured at λ_ex_ 355 nm and λ_em_ 460 nm using a microplate fluorescence reader (Synergy^®^ HTX multimode, BioTek Instruments, Inc., Winooski, VT, USA). The same reaction mixture without enzyme was used as a blank.

### 4.4. Immunofluorescence

The kidneys were excised, fixed in 4% paraformaldehyde-PBS, and dehydrated in ascending grades of ethanol, cleaned in xylene, and embedded in paraffin. The kidney sections were cut at 3 μm thickness and mounted in coverslips for immunostaining. The sections were incubated with blocking solution for 1 h at room temperature followed by incubation at 4 °C overnight with the following primary antibodies (1:500): nSMase (mouse monoclonal antibody, sc-166637, Santa Cruz Biotechnology, Dallas, TX, USA), nCDase—FITC (mouse monoclonal antibody, sc-374634 FITC, Santa Cruz Biotechnology), SphK1—FITC (mouse monoclonal antibody, sc-365401 FITC, Santa Cruz Biotechnology) [55]. Only the sections with nSMase were incubated with the secondary antibody (m-IgGκ BP-FITC (sc-516140, Santa Cruz Biotechnology) at 4 °C overnight.

To identify the presence of nSMase, nCDase, and SphK1 in the glomerulus and subsegments of the renal tubule, the tissues were co-stained with anti-thymocyte-1 (Thy-1 PE) (mouse monoclonal antibody, sc-53116 PE, Santa Cruz Biotechnology) as a marker of mesangial cells [56], anti-aquaporin-1 (AQP1) (mouse monoclonal antibody, sc-25287 PE, Santa Cruz Biotechnology) as a marker of proximal tubule and thin descending limb of Henle’s loop and the vasa recta in the outer medulla [57], anti-ClC-K1 (Santa Cruz Biotechnology) as a marker of thin ascending limb of Henle’s loop in inner medulla (tAL) [58], anti-Tamm–Horsfall protein (THP) (mouse monoclonal antibody, sc-271022 PE, Santa Cruz Biotechnology) as a marker of distal and thick ascending limb of Henle’s loop (TAL) [59], and anti-aquqporin-2 (AQP2) (mouse monoclonal antibody, sc-47710 AF594, Santa Cruz Biotechnology) as a marker of principal cells of the collecting duct [60,61]. Specificity of immunostaining was assessed by incubation in the absence of primary antibody and to reduce autofluorescence, we used TrueBlack Lipofuscin Autofluorescence Quencher (#23007; Biotium, TermoFisher, Waltham, MA, USA) [62]. Fluorescence images were obtained using a Cell Imaging Station (Life Technologies, Carlsbad, CA, USA).

### 4.5. Statistical Analysis

Statistical analysis was performed using Prism software (GraphPad 6, San Diego, CA, USA). Results are presented as means ± SE. The significance of differences between groups was evaluated via ANOVA followed by the Bonferroni post hoc test. Differences with *p* < 0.05 were considered statistically significant.

## 5. Conclusions

In conclusion, our results suggest that aSMase and nCDase activity in urine could be a novel predictor of early slight ultrastructural changes in the nephron, aSMase and nCDase as glomerular injury biomarkers, and nSMase as a tubular injury biomarker in diabetic and hypertensive rats.

## Data Availability

Data are contained within the article and supplementary materials.

## Supplementary Materials

The following supporting information can be downloaded at: https://www.mdpi.com/article/10.3390/ijms242316633/s1.
