# Supplementary figures and images for: Immunolocalization of Sphingolipid Catabolism Enzymes along the Nephron: Novel Early Urinary Biomarkers of Renal Damage
Source: Int J Mol Sci. 2023 Nov 23;24(23):16633. doi: 10.3390/ijms242316633 (PMC10706607; doi:10.3390/ijms242316633)

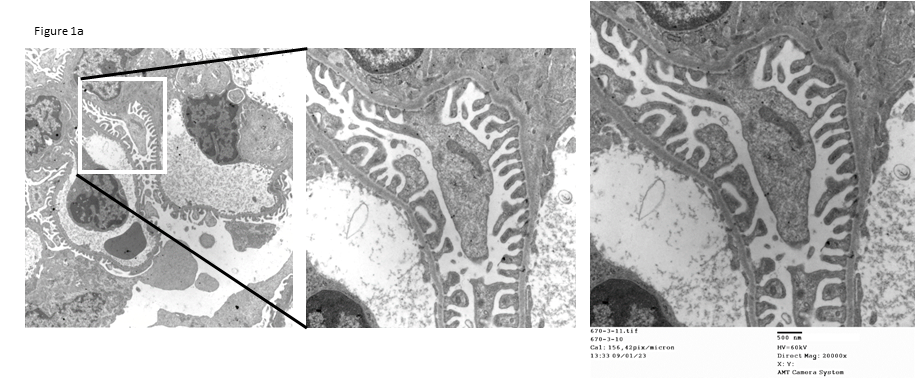

Supplement: Supplementary file 1 [file ijms-24-16633-s001.zip › Figure 1a.TIF]

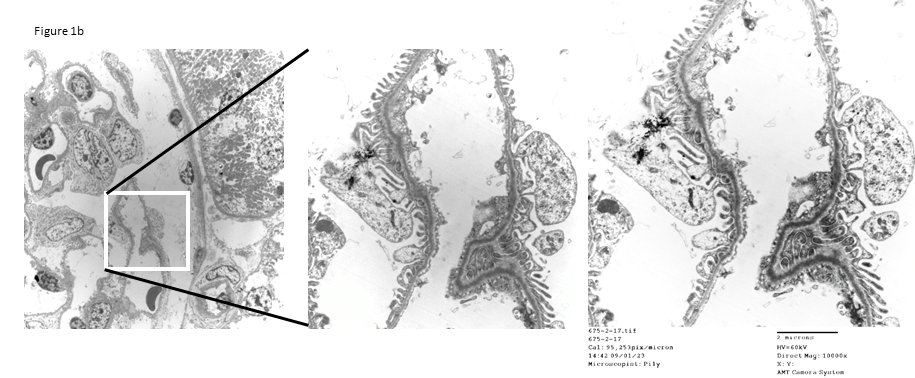

Supplement: Supplementary file 1 [file ijms-24-16633-s001.zip › Figure 1b.TIF]

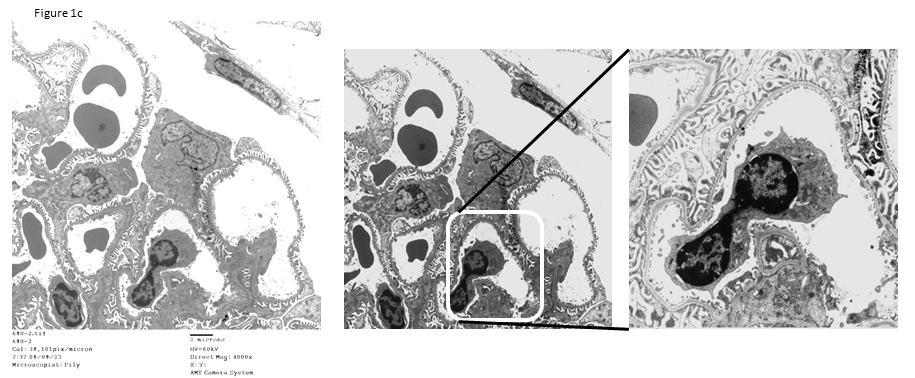

Supplement: Supplementary file 1 [file ijms-24-16633-s001.zip › Figure 1c.TIF]

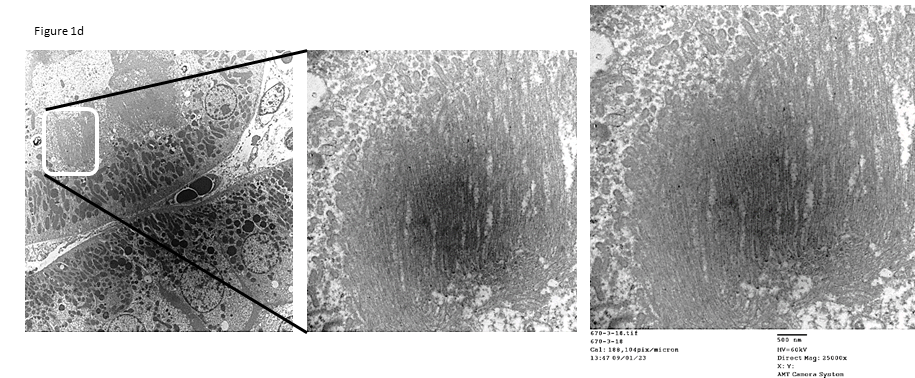

Supplement: Supplementary file 1 [file ijms-24-16633-s001.zip › Figure 1d.TIF]

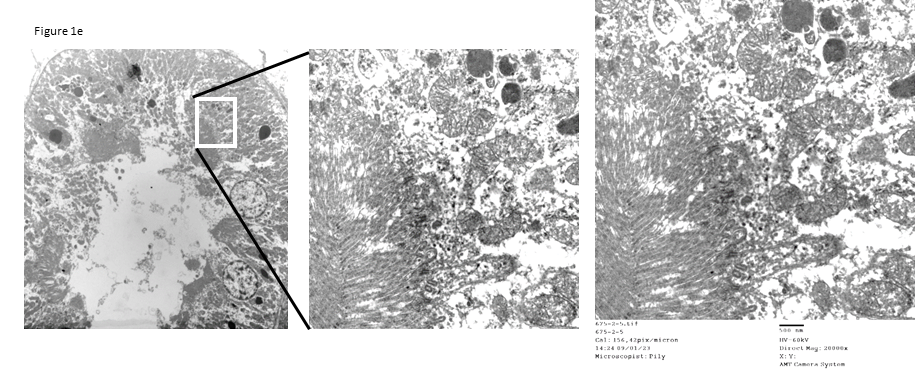

Supplement: Supplementary file 1 [file ijms-24-16633-s001.zip › Figure 1e.TIF]

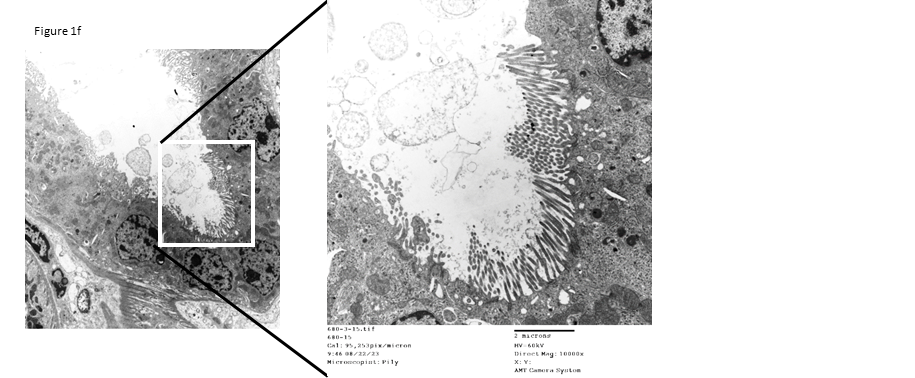

Supplement: Supplementary file 1 [file ijms-24-16633-s001.zip › Figure 1f.TIF]

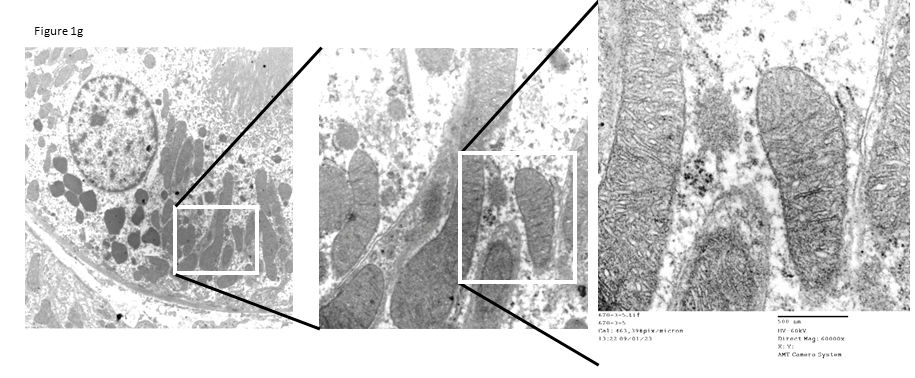

Supplement: Supplementary file 1 [file ijms-24-16633-s001.zip › Figure 1g.TIF]

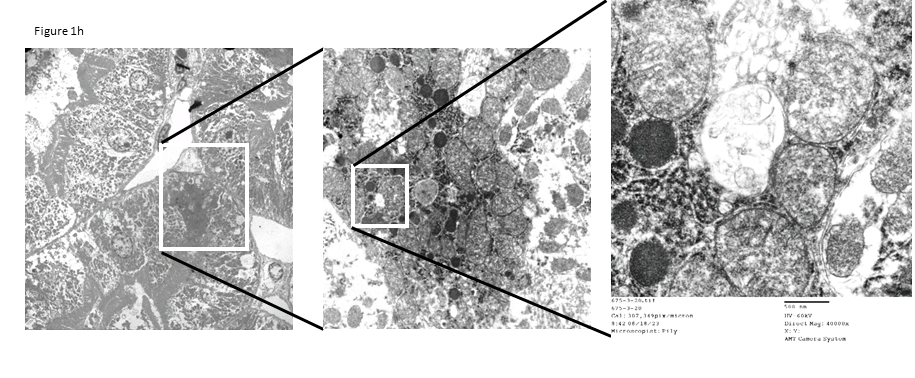

Supplement: Supplementary file 1 [file ijms-24-16633-s001.zip › Figure 1h.TIF]

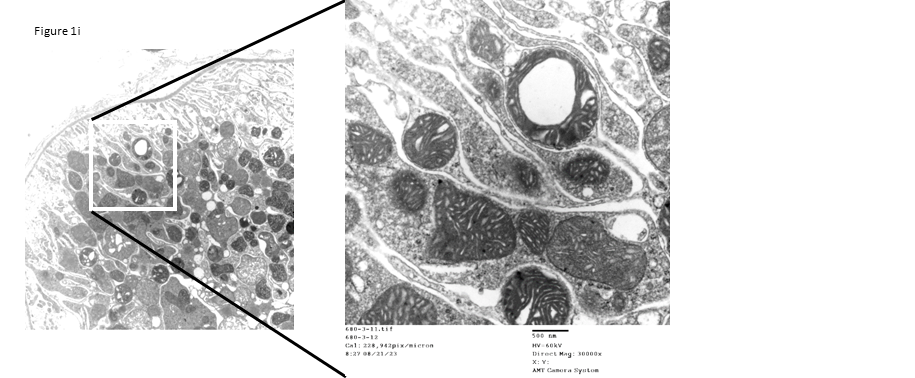

Supplement: Supplementary file 1 [file ijms-24-16633-s001.zip › Figure 1i.TIF]
